# 

**Published:** 1924-07

**Authors:** 


### RECENT APPOINTMENTS.

### Medical Officers, &c.

Bowes, G. K., M.D., D.P.H.; Assistant Medical Officer, Birmingham.

Caird, A. J., M.B., Ch.B.; Honorary Assistant Surgeon, The Infirmary, Cumberland.

Champneys, W. D., M.A., M.B., Ch.B., D.P.H., Clinical Assistant, Great Ormonde Street; Assistant Medical Officer, Willesden.

Falconer, K., M.B., Ch.B.; Medical Officer of Health, Drolysden.

Gray, Alice J., Internal Clinical Assistant, Infants' Hospital, Vincent Square; Resident Medical Officer, Municipal Hospital, Willesden.

Lawson, Miriam A., L.R C.P., M.R.C.S.; Resident Medical Officer, St. Mary's Hospital for Women and Children.

### Public Health and Hospital Appointments.

Allsop, Mr.; Sanitary Inspector, Urban District Council, Sunbury.

Budd, Annie; Superintendent (Queen Victoria's Jubilee Institute), East London.

Cosserley, A. E., R.R.C.; Matron, Hartley Hospital, Colne.

Coulthard, Mary E.; Assistant Superintendent (Queen Victoria's Jubilee Institute), West Riding Nursing Association.

Daglish, S. M., C.M.B.; Matron, Herzl Moser Hospital, Leeds.

Davis, A., C.M.B.; Matron, Victoria Cottage Hospital, Thame.

Johnson, G. M., C.M.B.; Matron, London Female Lock Hospital, Harrow.

Sampson, Gladys Assistant Matron, General Hospital, Wolverhampton.

Slack, Christina M.; Assistant Superintendent (Queen Victoria's Jubilee Institute), West Riding Nursing Association.

Thomas, Lily M.; Health Visitor, Ogmore and Garw Urban District.

Harris, Marjorie, M.B., Ch.B., D.P.H., Assistant Medical Officer, Oldham; Medical Officer, Manchester.

Brown, F. W. Campbell, M.D.; Medical Officer, Birkenhead.

Brown, Charles M., M.B., Ch.B.; Assistant Resident Medical Officer, City Isolation Hospital, Leicester.

Salmon, Elsie M., C.M.B., Assistant Matron, Royal Hospital, Chesterfield; Matron, County Sanatorium, Derbyshire.

Thompson, N., Assistant Matron, Horton Mental Hospital; Matron, Mental Hospital, Leavesden.

Walker, Mary Robertson; Lady Superintendent, Royal Children's Hospital, Manchester.

Willey, Dorcas; Matron, Cottage Hospital, Whitby.

Jackson, Dorothy Grierson, C.M.B.; Nurse, Welfare Centre, North Islington.

Maclachlan, Rachael, C.M.B.; County Superintendent and Supervisor, Infant Welfare Visitors, Morayshire.

### ANSWERS TO CORRESPONDENTS.

B.A.—The address of the Mental After-Care Association is Church House, Westminster. The Secretary is Miss Vickers.

T. B.—The National Association for the Prevention of Tuberculosis, 20 Hanover Square, W., probably publish something of the kind.

X.—No; there is no Ministry of Health in Ireland.

Hospital Sunday.—The Secretary, Hospital Sunday Fund, Mansion House, E.C., will send you particulars of how to organise a Hospital Sunday. Mr. S. R. Lamb, Joint Hospital Council, St. Peter's Close, Hartshead, Sheffield, will help you with schemes for appeals.

Arnold.—We believe a good glove sterilizer has recently been produced by Messrs. James Slater & Co., Ltd., of 50 and 51, Wells-street, W. 1.

Plaster.—Perhaps you have not tried in the right quarter. Apply to Messrs. Edmund Taylor, Ltd., of Salford, whose zinc oxide plasters may be thoroughly relied upon.

### Managerial Notices.

*All letters on Business matters must be addressed to the Manager, "The Hospital and Health Review," 28 and 29 Southampton Street, London, W.C. 2.*

RATES FOR SUBSCRIPTION (payable in advance).

| | | | |
|---|---|---|---|
| Three Months (including Postage) | .. .. | 1s. | 9d. |
| Six Months do. | .. .. | 3s. | 6d. |
| Twelve Months do. | .. .. | 7s. | 0d. |

**It is especially requested that remittances be made by Cheque or Postal Order and not Stamps.**

Subscriptions, which may commence at any time, are payable in advance. Cheques and Post-Office Orders should be crossed Westminster Bank, Covent Garden Branch, and made payable to the Manager of THE HOSPITAL AND HEALTH REVIEW.

**Advertisements.** Scale of charges and particulars of special positions may be obtained on application to the Manager.

*SUBSCRIPTION FORM.*

*Please send me* THE HOSPITAL AND HEALTH REVIEW *for.............. months, commencing with your issue of ..............., for which please find enclosed ........*

*Name* ..............................

*Address* ..............................

..............................

*Date* ..............................

*To*.... ..............................*Newsagent*

